# *E. coli* diversity: low in colorectal cancer

**DOI:** 10.1186/s12920-020-0704-3

**Published:** 2020-04-06

**Authors:** Le Tang, Yu-Jie Zhou, Songling Zhu, Gong-Da Liang, He Zhuang, Man-Fei Zhao, Xiao-Yun Chang, Hai-Ning Li, Zheng Liu, Zhi-Rong Guo, Wei-Qiao Liu, Xiaoyan He, Chun-Xiao Wang, Dan-Dan Zhao, Jia-Jing Li, Xiao-Qin Mu, Bing-Qing Yao, Xia Li, Yong-Guo Li, Li-Bo Duo, Li Wang, Randal N. Johnston, Jin Zhou, Jing-Bo Zhao, Gui-Rong Liu, Shu-Lin Liu

**Affiliations:** 1 Systemomics Center, College of Pharmacy, and Genomics Research Center (State-Province Key Laboratories of Biomedicine-Pharmaceutics of China), Harbin Medical University, 157 Baojian Road, Harbin, 150081 China; 2 HMU-UCCSM Centre for Infection and Genomics, Harbin Medical University, Harbin, China; 3 Departments of Ecosystems and Public Health, University of Calgary, Calgary, Canada; 4 Present address: Department of Immunology, Capital Medical University, Beijing, China; 5 Department of Epidemiology, Public Health School, Harbin Medical University, Harbin, China; 6 Department of Colorectal Surgery of the Second Affiliated Hospital, Harbin Medical University, Harbin, China; 7 Present address: Department of Colorectal Surgery, National Cancer Center/National Clinical Research Center for Cancer/Cancer Hospital, Chinese Academy of Medical Sciences and Peking Union Medical College, Beijing, China; 8 Department of Microbiology, Peking University Health Sciences Center, Beijing, China; 9 Microbiology, Immunology and Infectious Diseases, University of Calgary, Calgary, Canada; 10 Present address: Department of Clinical Neurosciences, University of Calgary, Calgary, Canada; 11 Translational Medicine Research and Cooperation Center of Northern China, Heilongjiang Academy of Medical Sciences, Harbin, China; 12 Department of Infectious Diseases of the First Affiliated Hospital, Harbin Medical University, Harbin, China; 13 Clinical Laboratory of Second Affiliated Hospital, Harbin Medical University, Harbin, China; 14 Biochemistry and Molecular Biology, University of Calgary, Calgary, Canada; 15 Department of Hematology of the First Affiliated Hospital, Harbin Medical University, Harbin, China

**Keywords:** *Escherichia coli*, Genetic diversity, Intestinal microbiota, Colorectal cancer, Longevity

## Abstract

**Background:**

*Escherichia coli* are mostly commensals but also contain pathogenic lineages. It is largely unclear whether the commensal *E. coli* as the potential origins of pathogenic lineages may consist of monophyletic or polyphyletic populations, elucidation of which is expected to lead to novel insights into the associations of *E. coli* diversity with human health and diseases.

**Methods:**

Using genomic sequencing and pulsed field gel electrophoresis (PFGE) techniques, we analyzed *E. coli* from the intestinal microbiota of three groups of healthy individuals, including preschool children, university students, and seniors of a longevity village, as well as colorectal cancer (CRC) patients, to probe the commensal *E. coli* populations for their diversity.

**Results:**

We delineated the 2280 fresh *E. coli* isolates from 185 subjects into distinct genome types (genotypes) by PFGE. The genomic diversity of the sampled *E. coli* populations was so high that a given subject may have multiple genotypes of *E. coli*, with the general diversity within a host going up from preschool children through university students to seniors. Compared to the healthy subjects, the CRC patients had the lowest diversity level among their *E. coli* isolates. Notably, *E. coli* isolates from CRC patients could suppress the growth of *E. coli* bacteria isolated from healthy controls under nutrient-limited culture conditions.

**Conclusions:**

The coexistence of multiple *E. coli* lineages in a host may help create and maintain a microbial environment that is beneficial to the host. As such, the low diversity of *E. coli* bacteria may be associated with unhealthy microenvironment in the intestine and hence facilitate the pathogenesis of diseases such as CRC.

## Background

*Escherichia coli* had been generally known as commensal bacterial components of the normal microbiota in the gastrointestinal tract of humans and animals until the 1940s, when a variety of pathogenic strains began to be reported [1]. Pathogenic *E. coli* have different types, causing intestinal (see a nice review in [2]) or extra-intestinal infections [3–6]. New pathogenic types of *E. coli*, such as *E. coli* strains associated with Crohn disease [7–9] as well as those associated with colorectal cancer (CRC) [10–17], have continually been reported. Bacteria in the *Shigella* genus are closely related to *E. coli* and have been treated as pathogenic branches of *E. coli* [18, 19]; *E. coli* and *Shigella* together are often referred to as *E. coli* complex bacteria. To date, a large number of pathogenic *E. coli* isolates have been extensively studied and categorized into phylogenetic groups or clonal complexes according to their genetic differences due largely to their clinical significance [20–23]. However, whether the commensals, as potential evolutionary origins of the emerged and emerging pathogens, consist of monophyletic or polyphyletic *E. coli* populations is unclear.

In this study, we collected 2280 *E. coli* isolates from fresh fecal specimens of healthy individuals in three age groups, who did not have intestinal or extra-intestinal illnesses, and of CRC patients for comparative studies. Based on their genomic differences, we delineated the bacteria into discrete genomic types (genotypes). In general, the analyzed *E. coli* isolates were highly diverse, with one genotype having from several hundred to over a thousand genes not found in any other types compared and a particular participant harboring from a single to multiple genotypes of *E. coli*. Remarkably, the diversity went up with age in the groups of healthy subjects, from preschool children through university students to seniors of a longevity village, but the CRC patients of all ages had the lowest diversity. These results suggest that the coexistence of multiple benign *E. coli* lineages in the same microbiota may help create a beneficial microbial environment for human health.

## Methods

### Bacterial strains

Fecal specimens were collected from 68 preschool children of Yifu Kindergarten, three to six years old; 87 university students of Harbin Medical University, 17 to 22 years old; 15 senior people of Bama Longevity Village, Guangxi Province, 90 to 106 years old; and 15 colorectal cancer (CRC) patients from the Second Affiliated Hospital of Harbin Medical University, 34 to 77 years old (Table 1). The healthy participants did not have intestinal or extra-intestinal illnesses, and the CRC patients were diagnosed by experts of the Second Affiliated Hospital of Harbin Medical University. The participants of all four groups did not use any antimicrobials in the past 6 months prior to specimen collection. We obtained a written informed consent from each participant or their guardian and the present work was approved by the Ethics Committee of Harbin Medical University. All experiments were performed in accordance with relevant guidelines and regulations, consistent with the 1975 Declaration of Helsinki. For isolating the bacteria, we spread 30 μl X-gal (20 mg/ml) on the LB plate [24] and purified the bacteria by streaking a single blue colony on fresh LB plates. Bacterial identification was performed at the Clinical Laboratory of the Second Affiliated Hospital, Harbin Medical University, and all bacterial strains were confirmed to be *E. coli* before analyses. The bacterial strains were stocked at -80 °C in 25% glycerol and streaked on an LB plate for another round of single colony isolation by incubation at 37 °C overnight prior to use.

**Table 1:** *E.coli* genotype profiles of participants

No.	Participant ID	Sex	Age	Number of Genotypes^a^
**Children**				
1	WZW	Male	5	1
2	ZYC	Male	5	2
3	WSC	Male	6	1
4	JZH	Female	5	1
5	XZM	Male	6	1
6	XHR	Female	6	1
7	SYF	Male	4	2
8	ZJH	Female	6	1
9	HJB	Male	5	2
10	CYF	Female	3	2
11	SRH	Male	3	2
12	LBW	Male	4	3
13	WYB	Female	3	1
14	WYH	Female	3	1
15	GB	Male	4	2
16	KK	Male	6	1
17	ZWH	Female	4	1
18	CEY	Female	4	2
19	HYX	Male	3	1
20	QMZ	Male	3	1
21	LMQ	Male	4	1
22	XWR	Female	6	1
23	YPY	Male	5	2
24	SBW	Male	4	2
25	ZAK	Female	4	3
26	LJY	Female	6	1
27	ZZJ	Male	5	3
28	XHS	Male	4	1
29	LZT	Male	5	1
30	HYH	Female	5	1
31	YZY	Female	5	1
32	JYH	Female	5	4
33	TEZ	Male	3	1
34	WYF	Female	5	1
35	XZH	Male	6	3
36	ZHL	Male	4	3
37	QJY	Male	6	1
38	XZX	Female	4	2
39	DYL	Female	4	2
40	YPY	Female	6	2
41	LTW	Male	5	3
42	CPY	Male	6	2
43	WJS	Male	6	1
44	HBY	Male	5	3
45	GRY	Female	3	2
46	LEQ	Female	3	2
47	LZY	Male	4	1
48	XWQ	Female	4	2
49	WZH	Male	6	3
50	JYX	Male	5	2
51	YCX	Female	3	1
52	ZJH	Female	3	1
53	PJY	Male	6	1
54	LXZ	Male	5	1
55	SYZ	Male	3	1
56	FSK	Female	3	1
57	WC	Male	3	2
58	RHM	Female	6	1
59	CBC	Female	4	1
60	ZMX	Female	5	2
61	WX	Female	3	1
62	WJT	Female	3	3
63	LXY	Female	4	2
64	YQT	Female	3	1
65	LZR	Male	3	2
66	TZL	Male	5	1
67	YZY	Female	5	1
68	ZYJ	Female	4	1
**Students**				
1	YZY	Female	18	2
2	WB	Male	17	5
3	YX	Male	17	2
4	DSJ	Female	21	1
5	AB	Male	19	5
6	TWX	Male	19	3
7	MP	Male	18	4
8	ZGQ	Female	20	6
9	YX	Male	20	7
10	WHY	Female	19	4
11	FCL	Female	20	3
12	HSJ	Female	19	2
13	ZJL	Female	20	4
14	GMQ	Female	22	6
15	ZSY	Female	21	1
16	TZL	Male	20	7
17	LL	Female	20	5
18	SMN	Female	20	3
19	LY	Female	19	3
20	YZR	Female	20	4
21	CPP	Female	21	4
22	CH	Male	21	1
23	WQY	Male	19	2
24	HQ	Female	19	1
25	FC	Male	22	3
26	ZS	Female	21	6
27	MWH	Female	19	4
28	SQ	Male	22	4
29	LSY	Female	18	3
30	YXX	Male	20	3
31	ZYX	Male	20	3
32	FYJ	Female	22	2
33	MHY	Female	21	7
34	XXM	Female	19	2
35	BJ	Female	18	2
36	CS	Male	21	4
37	SX	Female	21	1
38	CYB	Female	21	5
39	LHM	Female	21	2
40	LYY	Female	20	2
41	WRM	Female	19	2
42	WY	Male	19	2
43	LXL	Female	22	1
44	WYJ	Female	19	2
45	LHC	Male	20	7
46	SR	Female	20	3
47	HL	Female	21	2
48	CYJ	Female	22	1
49	WYT	Female	21	3
50	ZQZ	Female	22	1
51	ZJB	Male	21	4
52	ZWL	Male	20	1
53	WHR	Female	21	4
54	ZYZ	Male	19	6
55	WJJ	Female	19	4
56	QJ	Female	19	2
57	WYY	Female	18	2
58	LJ	Female	19	3
59	SN	Female	18	2
60	WSL	Female	21	5
61	SH	Male	20	4
62	LYY	Female	20	2
63	JHN	Male	20	4
64	YMX	Female	20	2
65	XKL	Female	19	3
66	XX	Female	20	3
67	WY	Male	20	2
68	YR	Female	19	3
69	MXL	Female	19	5
70	GS	Female	19	2
71	LZY	Female	19	2
72	TR	Female	19	2
73	SHY	Female	19	1
74	GYX	Female	21	3
75	WL	Female	19	2
76	LLJ	Female	19	1
77	SYH	Female	19	3
78	MZY	Female	19	1
79	BZ	Male	18	2
80	FXF	Female	20	3
81	HJ	Female	19	1
82	HKP	Male	20	1
83	FR	Female	18	2
84	JT	Female	18	1
85	WL	Female	19	4
86	PHM	Female	17	4
87	ZMM	Female	19	2
**Bama Seniors**				
1	BMS1	Female	106	5
2	BMS2	Female	90	5
3	BMS3	Male	91	5
4	BMS4	Female	105	6
5	BMS5	Female	102	4
6	BMS6	Female	96	4
7	BMS9	Male	93	4
8	BMS11	Female	100	5
9	BMS12	Female	101	5
10	BMS17	Male	94	5
11	BMS18	Female	103	13
12	BMS21	Female	98	13
13	BMS28	Male	92	5
14	BMS63	Female	93	3
15	BMS67	Female	96	8
**CRC patients**				
1	LSJ	Female	68	2
2	ZFQ	Female	63	1
3	LJH	Female	45	2
4	SSR	Male	75	1
5	CYZ	Male	61	1
6	LDP	Male	52	1
7	GJB	Male	56	1
8	WWX	Male	57	1
9	ZMD	Female	61	1
10	BZZ	Female	77	4
11	ZSH	Female	61	1
12	ZWF	Male	61	1
13	ZYM	Male	38	4
14	BZS	Male	64	3
15	CYT	Male	34	3

^a^ Number of gentypes over the number of strains analyzed: for seniors,16 colonies were analyzed; for participants of the other three groups, 12 colonies were analyzed

### Genomic comparisons by pulsed field gel electrophoresis techniques

Methods for intact genomic DNA extraction from the bacteria and PFGE analyses were according to the protocols published previously [25–27]. The endonuclease I-CeuI recognizes phylogenetic diversity of the bacteria from the genus and up levels [27–29] and cleavage data from the CTAG-recognizing endonucleases reflect bacterial diversity at the species level, which are consistent with genomic sequence data [30–33].

### Genomic sequencing and analysis

Genomic sequencing of the bacteria was conducted on the Illumina HiSeq 2000 platform, which produced 620 Mb data for each of the strains. Library construction and sequencing were carried out according to the manufacturer’s recommendation at the Illumina web site. The sequence data from Illumina HiSeq 2000 were assembled with SOAPdenovo 2.04 software and the sequence analysis was performed as previously described [34, 35]. The draft genome sequences can be accessed under accession numbers shown in Table 2. We predicted genes from the assembled sequences using Glimmer 3.02 [66, 67].

**Table 2:** Information of bacterial strains used in this study

Strain	Accession number	Pathogenicity/Source^a^	Reference
*E.coli* MG1655	NC_000913	Non pathogenic	[36]
*E.coli* H10407	NC_017633	ETEC	[37]
*E.coli* P12b	NC_017663	Non-pathogenic	[38]
*E.coli* UMNK88	NC_017641	Enterotoxigenic *E. coli* (ETEC)	https://www.ncbi.nlm.nih.gov/nuccore/NC_017641
*E. coli* REL606	CP000819	Non-pathogenic	https://www.ncbi.nlm.nih.gov/nuccore/CP000819
*E. coli* ATCC 8739	NC_010468		https://www.ncbi.nlm.nih.gov/nuccore/NC_010468
*E. coli* O78	NC_020163	APEC	[39]
*E. coli* E24377A	NC_009801	ETEC	https://www.ncbi.nlm.nih.gov/nuccore/NC_009801
*E. coli* 11,128	NC_013364	STEC/EHEC	[40]
*E. coli* 12,009	NC_013353	STEC/EHEC	[40]
*E. coli* SE11	NC_011415	Non pathogenic	[41]
*E.coli* IAI1	NC_011741	Non pathogenic	https://www.ncbi.nlm.nih.gov/nuccore/NC_011741
*E.coli* 2009EL-2050	NC_018650	EAggEC-EHEC	[42]
*E.coli* LY180	NC_022364		[43]
*E.coli* NA114	NC_017644	ExPEC (multidrug-resistant UPEC)	[44]
*E.coli* SE15	NC_013654	Non-pathogenic	[45]
*E.coli* E2348/69	NC_011601	EPEC	[46]
*E.coli* 536	NC_008253	ExPEC (UPEC)	[47]
*E.coli* UTI89	NC_007946	ExPEC (UPEC)	[48]
*E.coli* S88	NC_011742	ExPEC (neonatal meningitis)	[49]
*E.coli* LF82	NC_011993	AIEC	https://www.ncbi.nlm.nih.gov/nuccore/NC_011993
*E.coli* ED1a	NC_011745	Non-pathogenic	https://www.ncbi.nlm.nih.gov/nuccore/NC_011745
*E.coli* CFT073	NC_004431	ExPEC (UPEC)	[50]
*E.coli* UMN026	NC_011751	ExPEC (UPEC)	https://www.ncbi.nlm.nih.gov/nuccore/NC_011751
*E.coli* 042	NC_017626	EAEC	[51]
*E.coli* CE10	NC_017646	ExPEC (neonatal meningitis)	[52]
*E.coli* SMS-3-5	NC_010498	Multi-resistant	https://www.ncbi.nlm.nih.gov/nuccore/NC_010498
*E.coli* O55 RM12579	NC_017656	Atypical EPEC (aEPEC)	https://www.ncbi.nlm.nih.gov/nuccore/NC_017656
*E.coli* O55 CB9615	NC_013941	aEPEC	[53]
*E.coli* O157 Xuzhou21	NC_017906	STEC/EHEC	[54]
*E.coli* O157 Sakai	NC_002695	STEC/EHEC	[55]
*E.coli* O157 EDL933	NC_002655	EHEC	[56]
*Sh. boydii* Sb227	CP000036	Dysentery	[57]
*Sh. sonnei* 53G	NC_016822	Dysentery	https://www.ncbi.nlm.nih.gov/nuccore/NC_016822
*Sh. flexneri* 58,401	NC_008258	Dysentery	[58]
*Sh. flexneri* 2a 301	AE005674	Dysentery	https://www.ncbi.nlm.nih.gov/nuccore/AE005674
*Sh. dysenteriae* Sd197	NC_007606	Dysentery	[57]
*S. heidelberg* B182	NC_017623	*Salmonella* food poisoning	[59]
*S. typhimurium* LT2	NC_003197	Gastroenteritis	[60]
*S. paratyphi* B SPB7	NC_010102	Paratyphoid fever	https://www.ncbi.nlm.nih.gov/nuccore/NC_010102
*S. paratyphi* C RKS4594	CP000857	Paratyphoid fever	[35]
*S. choleraesuis* SC-B67	NC_006905	Systemic infections	[61]
*S. dublin* CT_02021853	NC_011205	Systemic infections	[62]
*S. enteritidis* P125109	NC_011294	Gastroenteritis	[63]
*S. pullorum* RKS5078	NC_016831	Chicken dysentery	[34]
*S. gallinarum* 287/91	NC_011274	Chicken typhoid fever	[63]
*S. paratyphi* A ATCC 9150	NC_006511	Paratyphoid fever	[64]
*S. typhi* Ty2	AE014613	Typhoid fever	[65]
*S. bongori* NCTC 12419	NC_015761	Pathogenic for cold-blooded animals	https://www.ncbi.nlm.nih.gov/nuccore/NC_015761
ccpm3961(AN61)	LLYC00000000	WZW, a healthy preschooler	This work
ccpm3962(AN62)	LLYD01000000	WZW, a healthy preschooler	This work
ccpm5062(AY62)	LLYE00000000	ZGQ, a healthy university student	This work
ccpm5063(AY63)	LLYF00000000	ZGQ, a healthy university student	This work
ccpm5064(AY64)	LLYG00000000	ZGQ, a healthy university student	This work
ccpm5065(AY65)	LLYH00000000	ZGQ, a healthy university student	This work
ccpm5069(AY69)	LLYI00000000	ZGQ, a healthy university student	This work
ccpm5071(AY71)	LLYJ00000000	ZGQ, a healthy university student	This work
ccpm5171(AZ71)	LLYK00000000	TZL, a healthy university student	This work
ccpm5172(AZ72)	LLYL00000000	TZL, a healthy university student	This work
ccpm5174(AZ74)	LLYM00000000	TZL, a healthy university student	This work
ccpm5175(AZ75)	LLYN00000000	TZL, a healthy university student	This work
ccpm5176(AZ76)	LLYO00000000	TZL, a healthy university student	This work
ccpm5177(AZ77)	LLYP00000000	TZL, a healthy university student	This work
ccpm5179(AZ79)	LLYQ00000000	TZL, a healthy university student	This work
BAMA0321	NPIO00000000	BMS9, a healthy longevity senior	This work
BAMA0315	NPIP00000000	BMS9, a healthy longevity senior	This work
BAMA0374	NPIQ00000000	BMS11, a healthy longevity senior	This work
BAMA0361	NPIR00000000	BMS11, a healthy longevity senior	This work
BAMA0397	NPIS00000000	BMS15, a healthy longevity senior	This work
ccpm6195	NQIN00000000	CRC-EC1, a CRC patient	This work
ccpm6201	NQIO00000000	CRC-EC1, a CRC patient	This work
ccpm6207	NQIP00000000	CRC-EC2, a CRC patient	This work
ccpm6219	NPIM00000000	CRC-EC3, a CRC patient	This work
ccpm6220	NPIN00000000	CRC-EC3, a CRC patient	This work

^a^ “Pathogenicity” for the reference *E. coli* complex and *Salmonella* strains, “source” for the 25 *E. coli* strains isolated for this study

### Phylogenetic analysis

Orthologs were determined by BLAST alignment with the criteria that identity was larger than 70% and alignment length was longer than 70% of the whole gene. Concatenation of conserved genes was done by using home-made Perl scripts. The phylogenetic tree was constructed by MEGA6.

### Detection of genetic boundaries

The detection of genetic boundaries was performed as previously described [68].

### Probing the pan-genomes

We determined the genes common to all compared strains and used the genes as the “core-genome” for the strains compared; we added all non-redundant genes of the bacterial strains in comparison to the core-genome to obtain the “pan-genome”. The analysis of pan-genomes and core-genomes was done by using home-made Perl scripts.

### Growth competition assays among the *E. coli* strains in different nutrient conditions

For the growth competition assays, we mixed three strains in a set, including one from a CRC patient and two from two separate healthy subjects. We inoculated a single colony from a strain into 4 ml LB broth and incubated the bacteria at 37 °C overnight. On day 1 of the growth competition assays, we transferred 1 ml of each of the three overnight cultures in a set into one fresh 15 ml test tube with a water-tight cap (for genomic DNA extraction manipulations [25];). After brief vortex mixing, we transferred an aliquot of 30 μl into a 10 ml test tube containing 3 ml LB broth and another aliquot of 30 μl into a 10 ml test tube containing 3 ml M9 medium [69] and incubated the 100-fold diluted cultures overnight at 37 °C with shaking (200 rpm); all ensuing cultures were conducted under such conditions. Then we extracted genomic DNA of the bacteria from each of the three single overnight cultures and the pooled cultures 1 ml each from the three single overnight cultures by procedures provided previously [25].

On day 2 of the assays, the overnight cultures initiated on day 1 were 100-fold diluted again (a 30 μl aliquot into a 10 ml test tube containing 3 ml LB broth and a another aliquot of 30 μl into a 10 ml test tube containing 3 ml M9 medium). This procedure was repeated daily until day 10, when a 30 μl aliquot from the day 10 LB culture was transferred into a 10 ml test tube containing 3 ml LB broth as usual and the 30 μl aliquot from the day 10 M9 culture was transferred into a 10 ml test tube also containing 3 ml LB broth, not M9 medium, to obtain sufficient bacterial cells for genomic DNA extraction on day 11.

The procedure of dilution and culture was terminated on day 11 and genomic DNA was extracted from the bacteria of the end cultures.

### Statistical analysis

Statistical analysis was conducted by using SAS version 9.1 (SAS Institute Inc., Cary, NC, USA) and GraphPad Prism statistical software; as the data were not normally distributed, Wilcoxon rank-sum test was used.

## Results

### Genomic diversity of *E. coli* in healthy people and CRC patients

**Fig. 1:**
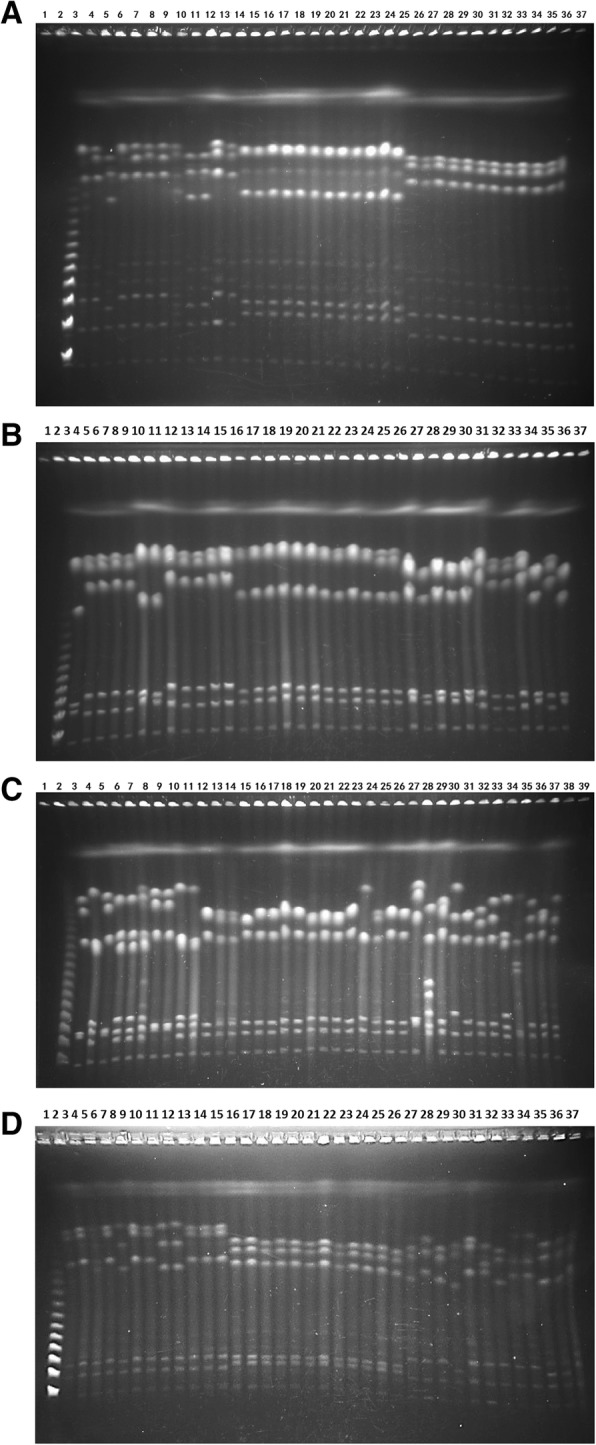
PFGE patterns of *E. coli* strains from four participant groups**. a** Preschool children (3–6 years old). Lanes: 1, Concatenated λ DNA as molecular size marker; 2, ccpm4786; 3, ccpm4787; 4, ccpm4788; 5, ccpm4789; 6, ccpm4790; 7, ccpm4791; 8, ccpm4792; 9, ccpm4793; 10, ccpm4794; 11, ccpm4795; 12, ccpm4796; 13, ccpm4797; 14, ccpm4798; 15, ccpm4799; 16, ccpm4800; 17, ccpm4801; 18, ccpm4802; 19, ccpm4803; 20, ccpm4804; 21, ccpm4805; 22, ccpm4806; 23, ccpm4807; 24, ccpm4808; 25, ccpm4809; 26, ccpm4834; 27, ccpm4835; 28, ccpm4836; 29, ccpm4837; 30, ccpm4838; 31, ccpm4839; 32, ccpm4840; 33, ccpm4841; 34, ccpm4842; 35, ccpm4843; 36, ccpm4844; 37, ccpm4845. **b** University students (17–22 years old). Lanes: 1, Concatenated λ DNA as molecular size marker; 2, ccpm5554; 3, ccpm5555; 4, ccpm5556; 5, ccpm5557; 6, ccpm5558; 7, ccpm5559; 8, ccpm5560; 9, ccpm5561; 10, ccpm5562; 11, ccpm5563; 12, ccpm5564; 13, ccpm5565; 14, ccpm5566; 15, ccpm5567; 16, ccpm5568; 17, ccpm5569; 18, ccpm5570; 19, ccpm5571; 20, ccpm5572; 21, ccpm5573; 22, ccpm5574; 23, ccpm5575; 24, ccpm5576; 25, ccpm5577; 26, ccpm5578; 27, ccpm5579; 28, ccpm5580; 29, ccpm5581; 30, ccpm5582; 31, ccpm5583; 32, ccpm5584; 33, ccpm5585; 34, ccpm5586; 35, ccpm5587; 36, ccpm5588; 37, ccpm5589. **c** Senior individuals (90–106 year old). Lanes: 1, Concatenated λ DNA as molecular size maker; 2, 9-MK1; 3, 9-MK2; 4, 9-MK3; 5, 9-MK4; 6, 9-MK5; 7, 9-MK6; 8, 9-MK7; 9, 9-MK8; 10, 9-CB1; 11, 9-CB2; 12, 9-CB3; 13, 9-CB4; 14, 9-CB5; 15, 9-CB6; 16, 9-CB7; 17, 9-CB8; 18, 11-MK1; 19, 11-MK2; 20, 11-MK3; 21, 11-MK4; 22, 11-MK5; 23, 11-MK6; 24, 11-MK7; 25, 11-MK8; 26, 11-CB1; 27, 11-CB2; 28, 11-CB3; 29, 11-CB4; 30, 11-CB5; 31, 11-CB6; 32, 11-CB7; 33, 11-CB8; 34, 15-MK1; 35, 15-MK2; 36, 15-MK3; 37, 15-MK4; 38, 15-MK5; 39, 15-MK6. **d** CRC cancer patients (34–77 years old). Lanes: 1, Concatenated λ DNA as molecular size maker; 2, ccpm6546; 3, ccpm6547; 4, ccpm6548; 5, ccpm6549; 6, ccpm6550; 7, ccpm6551; 8, ccpm6552; 9, ccpm6553; 10, ccpm6554; 11, ccpm6555; 12, ccpm6556; 13, ccpm6557; 14, ccpm6558; 15, ccpm6559; 16, ccpm6560; 17, ccpm6561; 18, ccpm6562; 19, ccpm6563; 20, ccpm6564; 21, ccpm6565; 22, ccpm6566; 23, ccpm6567; 24, ccpm6568; 25, ccpm6569; 26, ccpm6570; 27, ccpm6571; 28, ccpm6572; 29, ccpm6573; 30, ccpm6574; 31, ccpm6575; 32, ccpm6576; 33, ccpm6577; 34, ccpm6578; 35, ccpm6579; 36, ccpm6580; 37, ccpm6581

To probe the general diversity of commensal *E. coli* in the human microbiota and test for any possible associations between the diversity and health status, we made genomic comparisons on the 2280 *E. coli* isolates (see details in Methods and Table 1). To reveal the overall genomic differences among the *E. coli* strains, we profiled the endonuclease cleavage patterns of the bacteria using the pulsed field gel electrophoresis (PFGE) techniques, which reflect relatedness of the bacteria and can delineate very closely related bacteria into distinct phylogenetic lineages [28, 31]. We found that the *E. coli* strains had highly diverse cleavage patterns; particularly, the endonuclease I-CeuI delineated the *E. coli* strains into distinct genotypes (Fig. 1). I-CeuI cleaves a 26 bp sequence in the 23S rRNA genes [70, 71], which are evolutionarily conserved in both genomic location and nucleotide sequence, so its cleavage patterns reflect genomic distribution of the 23S rRNA genes and hence the overall physical structure of the bacterial genome [27, 28].

**Fig. 2:**
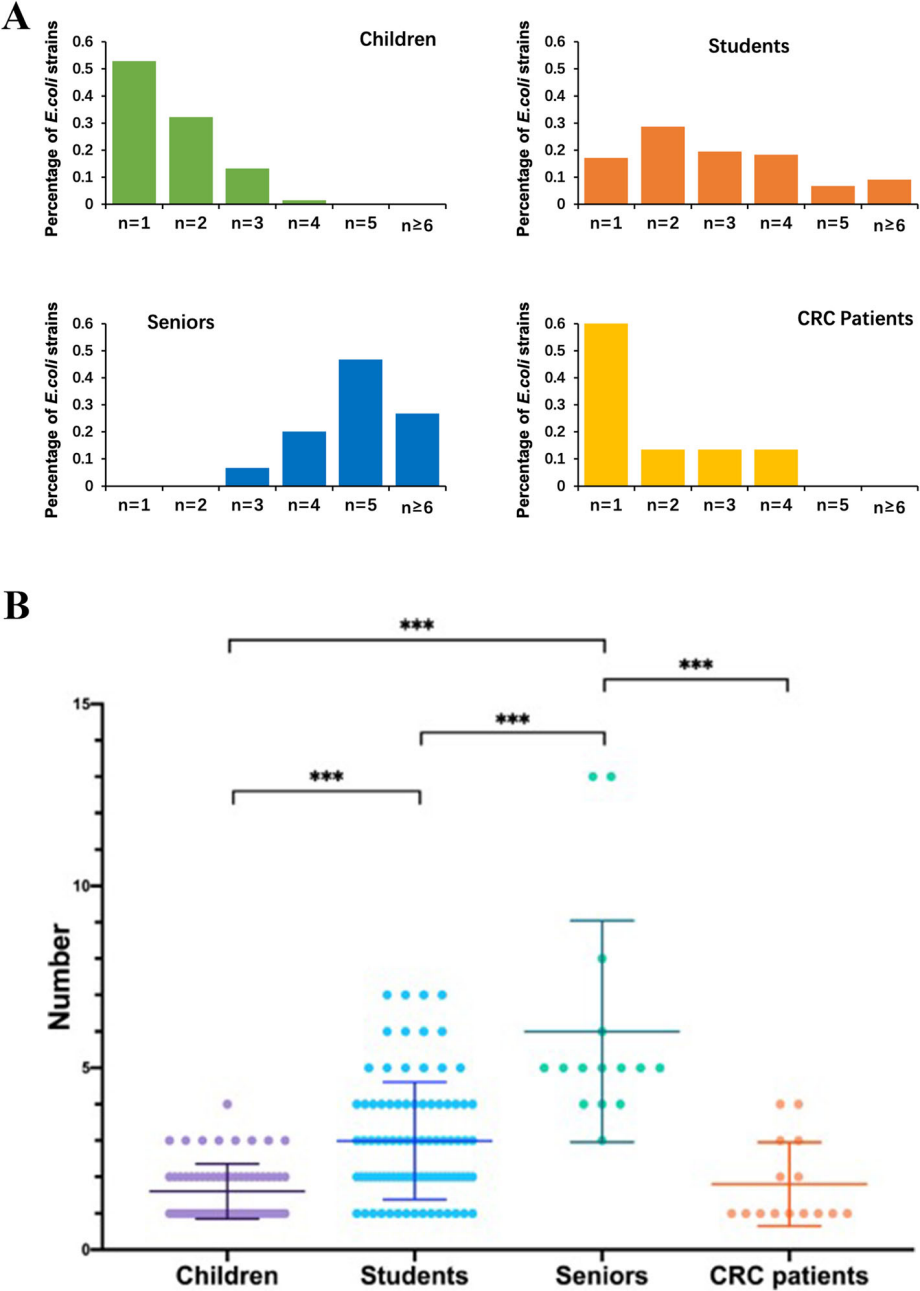
Diversity of *E. coli* in different age and health status groups. **a** Levels of *E. coli* diversity in the individual groups. The diversity is illustrated by percentages of participants in a group that have one, two or more genomic types among the *E. coli* strains analyzed. **b** Statistical comparisons of *E. coli* diversity among the four groups. ***: *p* < 0.0001 (Children vs Students, *p*-value = 3.291e-09; Children vs Seniors, p-value = 3.104e-10; Students vs Seniors, p-value = 7.226e-06; Seniors vs CRC patients, p-value = 6.83e-06; Students vs CRC patients, p-value = 0.0039; Children vs CRC patients, p-value = 0.8847.). Note that most CRC patients had only one genotype and most senior individuals had five or more genotypes

Although in general the *E. coli* isolates from the 185 participants had high genomic diversity, the level of diversity was different considerably among the groups: from very low to remarkably high among the individual participants (see Table 1). Notably, the magnitude of diversity went up with age in the healthy participants from preschool children through university students to senior individuals, whereas the colorectal cancer patients all had low diversity and the differences were statistically significant (Fig. 2). As shown in Table 1, as many as 13 genome types were resolved from the 16 randomly picked *E. coli* isolates of a senior individual, which suggests that higher diversity might be detected if more isolates from a subject were examined. To look into this, we extended the number of isolates from 12 to 16 per person to 60 in a subset of the subjects for the analysis; however, no further diversity was detected (data not shown).

### Phylogenetic divergence of the *E. coli* strains

**Fig. 3:**
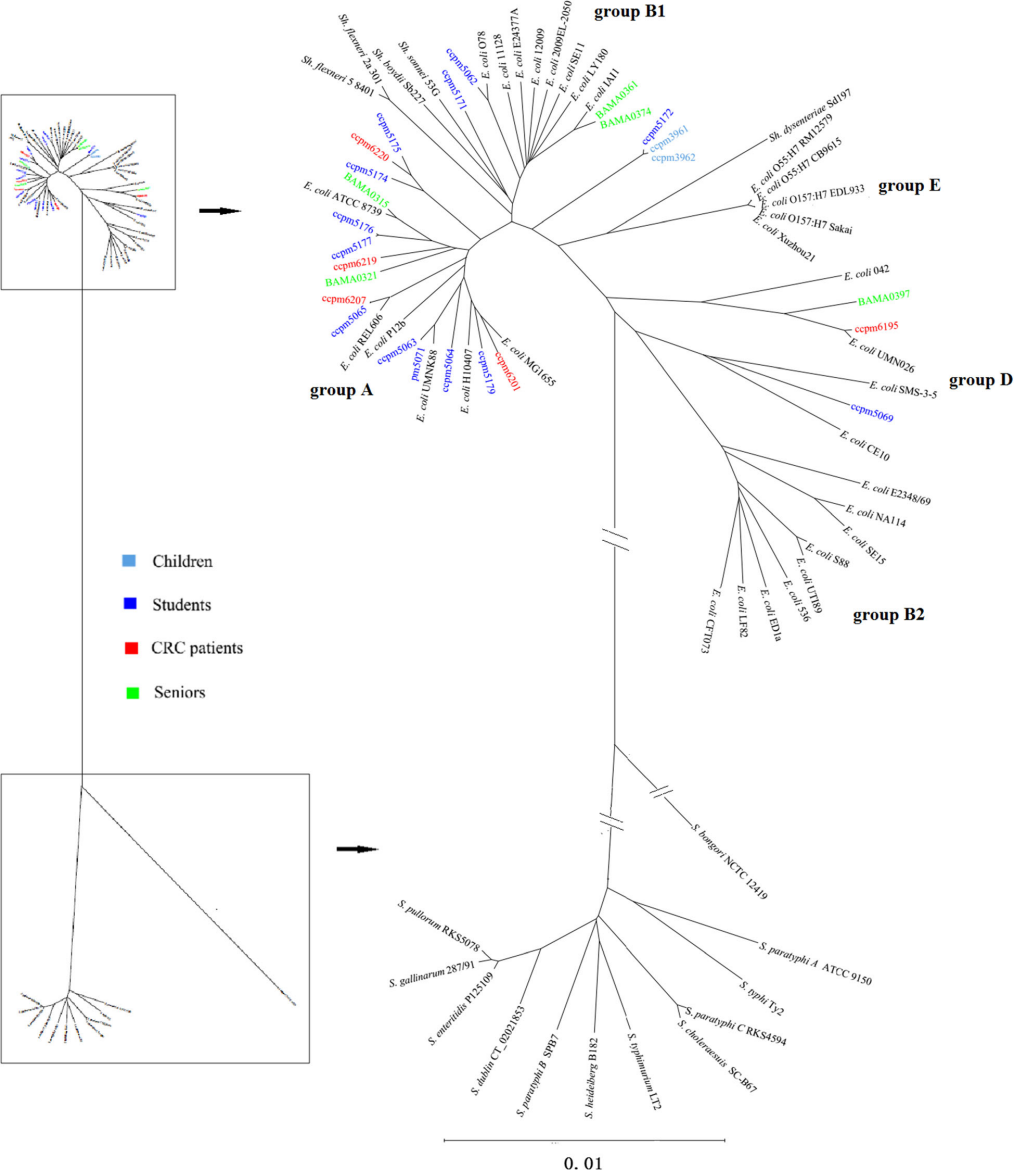
Phylogenetic tree for comparisons of relative genetic distances among the bacteria. On the left is a phylogenetic tree for strains of both *E. coli* and *Salmonella* and on the right is an enlarged part of the phylogenetic tree for *E. coli*. The tree was constructed with 1000 bootstrap replicates by the neighbor-joining (NJ) method

The high diversity of the analyzed *E. coli* strains as reflected by their I-CeuI profiles suggests different gene contents among the *E. coli* bacteria. To look into this, we sequenced 25 selected strains to make genomic comparisons at higher resolution and determine the phylogenetic relationships among them. We constructed a phylogenetic tree for the 25 *E. coli* strains along with 37 reference *E. coli* complex strains, representing phylogenetic groups A, B1, B2, D, E and *Shigella* lineages, as well as 12 reference *Salmonella* strains, representing the leading pathogens (Table 2), by concatenating and comparing 927 genes common to them. We included these reference *E. coli* and *Salmonella* strains in the phylogenetic analysis to estimate the evolutionary relationships as well as the genetic distances among the *E. coli* strains. On the constructed tree, the 25 *E. coli* strains were either mixed with strains of a phylogenetic group of *E. coli* (ccpm6201, ccpm5179, ccpm5064, ccpm5071, ccpm5063, ccpm5065, ccpm6207, ccpm6219, ccpm5177, ccpm5176, ccpm5174, ccpm6220, ccpm5175, BAMA0321 and BAMA0315 with Group A; ccpm5062, BAMA0361 and BAMA0374 with Group B1; and ccpm5069, ccpm6195 and BAMA0397 with Group D) or formed a branch between the groups (ccpm5172, ccpm3961 and ccpm3962 between Groups B1 and E). Additionally, ccpm5171 was clustered together with *Shigella*; none of the 25 strains was found to cluster with Group B2, which contains many extra-intestinal pathogens [72–74], or E, which contains O157:7 and O55:H7 [56] (Fig. 3). Also as shown in Fig. 3, the six strains of subject ZGQ and the seven strains of subject TZL (see Table 1 for details about subjects ZGQ and TZL) were broadly distributed on the phylogenetic tree without a clear tendency of clustering toward their origin hosts. On the other hand, some *E. coli* strains isolated from different hosts were closely related, such as ccmp3961 and ccpm3962 from WZW with ccpm5172 from TZL, and ccpm5065 from ZGQ with ccpm6207 from CRC-EC2, demonstrating the spreading of *E. coli* strains among the human populations (Fig. 3). Notably, genetic distances among the *E. coli* strains (including reference *E. coli* complex strains and the 25 *E. coli* strains isolated in this study) were similar to, or even greater than, those seen among the *Salmonella* lineages, indicating their phylogenetic divergence over evolutionary times.

### Genetic boundaries among the *E. coli* lineages

Such a high genetic diversity of the commensal *E. coli* isolates and the remarkable evolutionary distances among them suggest the existence of genetic boundaries between them to separate them into discrete phylogenetic clusters. To detect such postulated genetic boundaries among the *E. coli* bacteria, we determined the ratios of homologous genes that have identical nucleotide sequences between pairs of strains by the method as reported previously for *Salmonella* [75]. The overall scales of differences among the profiled ratios (Supplementary Table 1) were consistent with the relative genetic distances revealed on the phylogenetic tree among the bacteria (see Fig. 3). Similar to the *Salmonella* lineages [68, 75], the *E. coli* strains well separated on the phylogenetic tree had low ratios of homologous genes with zero nucleotide sequence degeneracy between them, mostly below 10% like in *Salmonella* (Supplementary Table 1), demonstrating the existence of genetic boundaries that circumscribe the bacteria into discrete clusters. Such remarkable genomic divergence among the *E. coli* strains suggests that they may also have large numbers of genes different from one another, analysis of which may lead to novel insights into the evolution of different *E. coli* lineages*,* especially regarding the emergence of nascent pathogens from their commensal ancestors.

### Profiling novel genes and probing the potential pan-genome

We annotated the genomes of the 25 sequenced *E. coli* strains. We first identified genes that are also present in strain K12 MG1655 (Supplementary Table 2), and then profiled genes that are not present in MG1655 nor in one or more of the 25 *E. coli* isolates (Supplementary Table 3 and Supplementary Figure 1). We found that a given strain may have hundreds of genes not present in other *E. coli* strains, further demonstrating enormous diversity of the commensal *E. coli* populations. Such large numbers of specific genes will certainly make an *E. coli* strain biologically distinct from other *E. coli* lineages, especially in terms of their contributions to the human health, including their potentials to benefit the host or to rise as novel pathogens. In any case, a plastic genomic construction would be a prerequisite for the *E. coli* bacteria to readily accept foreign genes and become a unique lineage.

**Fig. 4:**
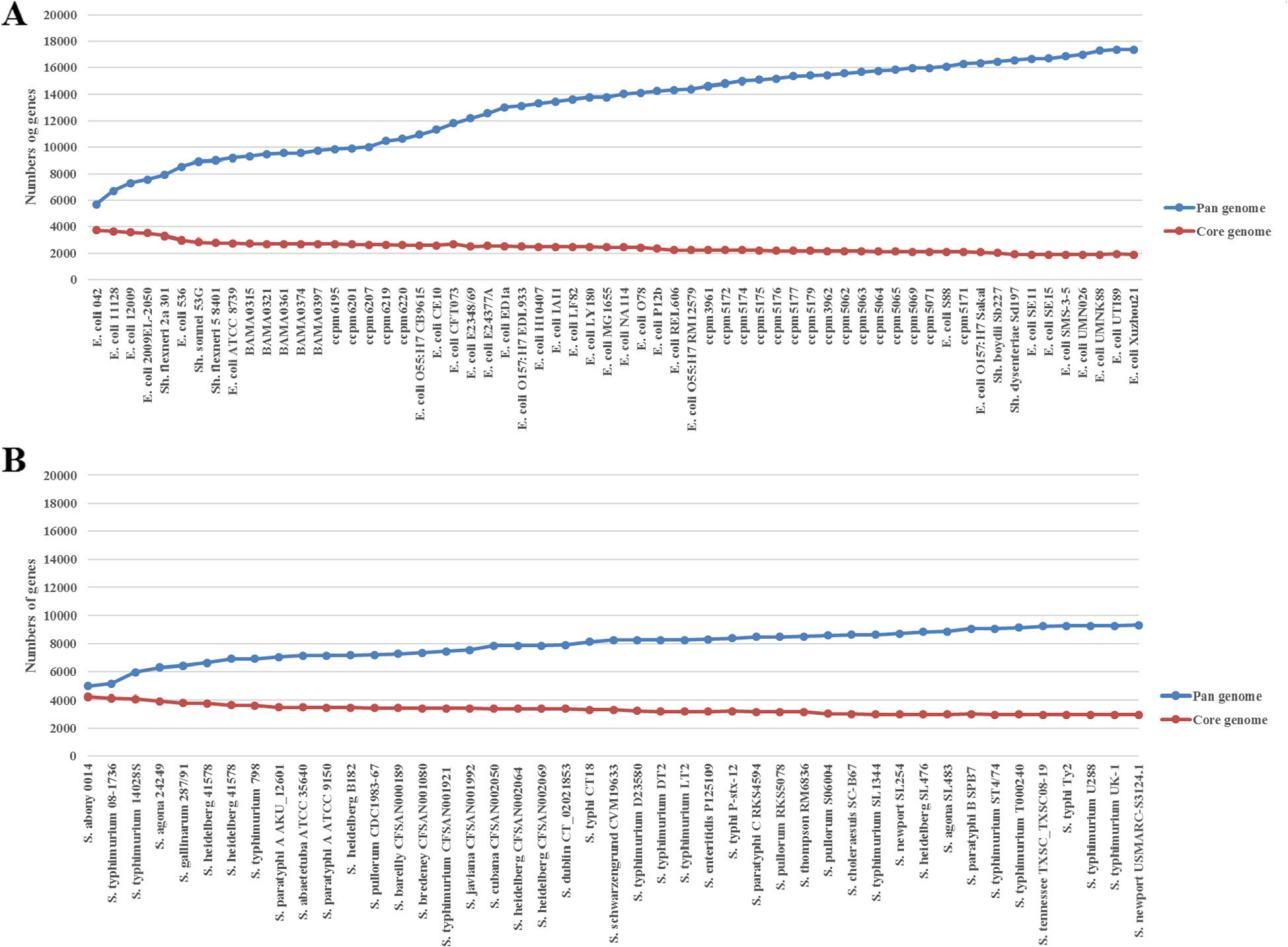
Comparison of sampled pan-genomes between *E. coli* and *Salmonella*. **a** Sampled pan-genome of *E. coli*; **b** Sampled pan-genome of *Salmonella*. Note that the *E. coli* pan-genome size keeps growing when new strains are included in the analysis

To validate the postulation that the *E. coli* genome is more amenable than those of some closely related bacteria to accept lateral genes and diverge, we probed the pan-genomes of *E. coli* and *Salmonella* and compared them. Comparison of 62 *E. coli* complex strains (including the 25 fresh *E. coli* isolates and the 37 reference *E. coli* complex strains) and 45 representative *Salmonella* strains revealed remarkable differences in their pan-genomes: whereas the 45 *Salmonella* strains had 2959 shared genes and 9338 total non-redundant genes, the 52 *E. coli* complex strains had only 1884 shared genes but as many as 17,335 total non-redundant genes (Fig. 4), giving an impression of an “open pan-genome” that may keep growing in size by accepting additional novel genes.

### Low *E. coli* diversity in colorectal cancer patients

The apparent tendency of low *E. coli* diversity in CRC patients pointed to a possibility of the bacteria as potential pathogens, directly or indirectly contributing to CRC, such as by creating an unhealthy intestinal microenvironment through suppressing or even purging beneficial *E. coli* lineages. To test such a postulation, we set up a series of growth competition assays and inspected the *E. coli* strains isolated from CRC patients for their competing growth abilities in rich (LB broth) or nutrient-limited (M9) medium with *E. coli* strains isolated from healthy subjects.

**Fig. 5:**
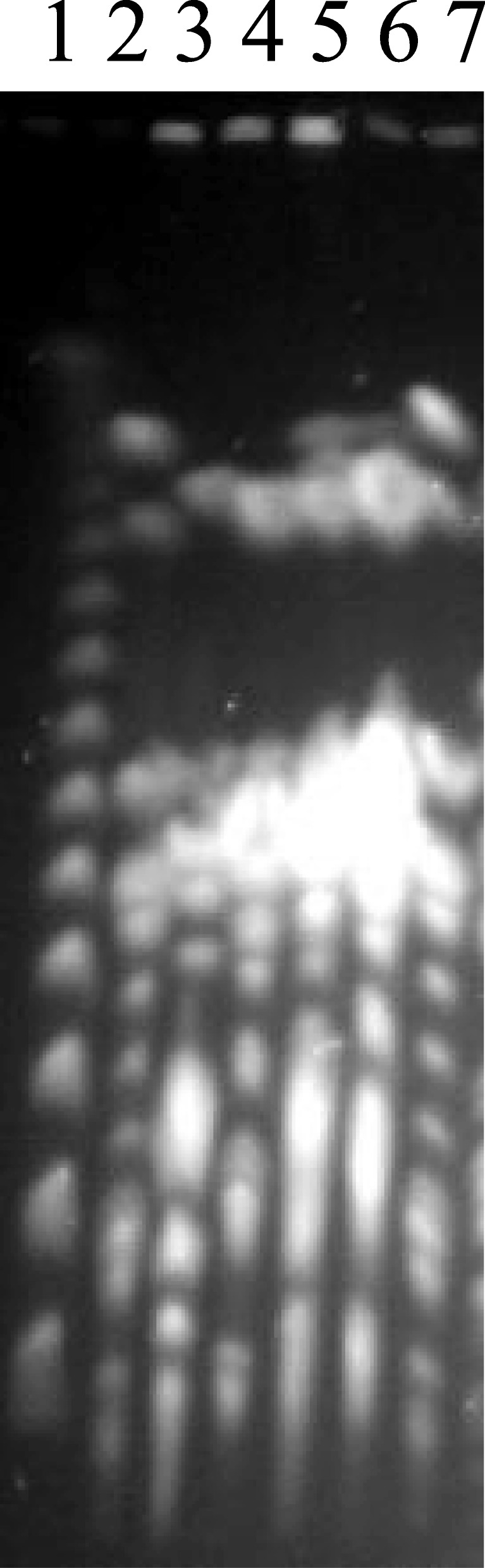
Growth competition among *E. coli* isolates from CRC patients and healthy controls. Lanes: 1, λ ladder as molecular size marker; 2, ccpm6195; 3, ccpm5172; 4, ccpm5062; 5, a mixture of ccpm6195, 5172 and 5062 before the competition assay; 6, a mixture of ccpm6195, 5172 and 5062 cultured in LB broth at the end of the competition assay; 7, a mixture of ccpm6195, 5172 and 5062 cultured in M9 medium at the end of the competition assay. Remarkably, after culture for 10 days in M9 medium, only ccpm6195 (see lane 7, the *E. coli* strain from a CRC patient, in which the genomic cleavage pattern is indistinguishable with that in lane 2) survived. These growth competition assays demonstrate that when nutrient was ample (as in LB broth), the three *E. coli* strains did not interfere with one another for growth; but when the nutrient was limited, the *E. coli* strains from a CRC patient had greater capabilities to compete for nutrient to grow

In such experiments, we co-cultured an *E. coli* strain isolated from a CRC patient with two *E. coli* strains isolated from two separate healthy subjects and detected the genomic cleavage patterns by the CTAG tetra-nucleotide sequence recognizing endonuclease XbaI in the end co-cultures (See details in Methods). XbaI and other CTAG recognizing endonucleases, such as BlnI/AvrII, SpeI and NheI, have rare cleavage sites in *E. coli* and the overall cleavage patterns are unique to bacteria of a particular phylogenetic cluster [26, 31, 76, 77]. In experiment set 1, for example, we included an *E. coli* strain from a CRC patient (ccpm6195) and one strain each from two university students (ccpm5172 and ccpm5602). After 10 days of diluted cultures by daily 100-fold dilution of 30 μl overnight culture into 3 ml fresh medium, we found that the three *E. coli* strains grew equally well in LB broth but the situation was dramatically different in M9 medium (Fig. 5). As shown in Fig. 5, the *E. coli* strain from a CRC patient (ccpm6195; lane 2) and the two *E. coli* strains from two university students (ccpm5172 and ccpm5602; lane 3 and 4, respectively) had distinct XbaI cleavage patterns, so they could be distinguished unambiguously. The mixture of the three strains showed all bandings of lanes 2, 3 and 4 on day 0 (Fig. 5, lane 5) when they were mixed immediately prior to genomic extraction. After incubation for 10 days in LB broth, the end mixture culture showed a similar growth pattern (Fig. 5, lane 6) to that of the initial mixture (Fig. 5, lane 5), whereas the end mixture culture of the three strains in M9 medium showed only the XbaI cleavage pattern of ccpm6195 (the *E. coli* strain from a CRC patient; Fig. 5, lane 7), demonstrating that ccpm6195 may have greater capability of harnessing the hardly available nutrient in the M9 medium, although direct or indirect suppression of the *E. coli* strains from healthy individuals by the *E. coli* strain from a CRC patient cannot be ruled out.

## Discussion

In this study, we demonstrated the associations of high phylogenetic diversity of *E. coli* with health, with the diversity going up with age from children through young adults to longevity seniors. We believe that the assumed commensal *E. coli* bacteria, i.e., those that are not associated with apparent intestinal or extra-intestinal infections, are the products of co-evolution with the human host, adapting to different microenvironments in the human intestine and providing a variety of beneficial functions to their host. Low diversity therefore may mean the absence of several such beneficial functions, making the host vulnerable to certain unhealthy factors and hence susceptible to some diseases such as CRC. Although the observed association between low *E. coli* diversity and CRC does not exclude the possibility that the conditions that lead to low *E. coli* diversity are conditions that lead to colorectal cancer as having been extensively discussed with a focus on changes in the intestinal microbiome [78, 79], we are inclined to believe low *E. coli* diversity, caused by the purging capabilities of certain non-benign *E. coli* lineages, to be a novel risk factor for CRC based on the results we obtained in this study, especially the phylogenetically distinct and biologically aggressive *E. coli* lineages.

These findings provoke a key question: what is the nature of the diversity? Are the diverse *E. coli* strains some randomly picked members of a wide spectrum of continual genetic variants collectively called a species of *E. coli* or do they represent discrete phylogenetic clusters? For a definite answer to this health-relevant question, we analyzed the collected *E. coli* isolates and dissected the diversity among them. The collected 2280 *E. coli* isolates were all assumed to be commensals, because they were not related to intestinal or extra-intestinal illnesses in the healthy participants. At least for this study, we also deemed the *E. coli* isolates from the CRC patients to be commensals, because etiological associations of *E. coli* with CRC had not been established.

Using a combination of genomic analysis methods, including PFGE to determine the global physical structure of the bacterial genome [25], CTAG tetra-nucleotide profiling by the use of CTAG sequence recognizing endonucleases to distinguish different phylogenetic clusters [80, 81], and whole genome sequence comparisons to identify genes common to the bacteria in comparison and those present only in a subset of the bacteria, we delineated these bacteria into discrete genotypes. It was the use of the combined methods that made this work possible. One of the core techniques used in this study was I-CeuI cleavage profiling by PFGE, which can categorize bacteria into genus or sub-genus phylogenetic levels unambiguously and intuitively [27, 28, 82]. Although the next generation sequencing techniques have been developing extremely rapidly, with continuously increasing capacity and radically dropping cost, sequencing of 2280 strains is still an enormous task, let alone the fact that all the thousands of bacterial genomes would have to be completely finished without a single gap or sequencing error for this kind of global genome comparisons. Additionally, distinguishing three co-cultured *E. coli* strains without labeling any of them by additional manipulations such as antimicrobial resistance markers can be conveniently and unambiguously achieved only by CTAG tetra-nucleotide sequence profiling; in fact, no any other molecular methods currently available can fulfill this task.

All the results demonstrate that the great diversity revealed among the 2280 *E. coli* strains was due to the divergence of the bacteria into discrete phylogenetic clusters, not a continual spectrum of genetic variants, over evolutionary times, during which the bacteria in the phylogenetic clusters accumulated genomic variations independently and became genetically isolated from one another by genetic boundaries. The absence of genetic continuum, or rather continuum of genetic variations, among the highly related *Salmonella* subgroup I pathogens has been previously proven [32, 33, 68, 75], but it is the first time for the *E. coli* bacteria to be delineated into independent phylogenetic lineages that did not have detectable continuum of genetic variations among them. Therefore, we think it reasonable to treat different genotypes of *E. coli* as different bacteria or as bacteria of highly related but distinct species, especially considering the finding that a given *E. coli* strain may have hundreds of genes not found in any other *E. coli* strains analyzed in this study (see Supplementary Table 3). As such, the commonly assumed commensal *E. coli* bacteria with large numbers of genes specific to only one or a few genotypes may accomplish a wide range of different biological activities for the benefit of their host and lack of any of them may mean the lack of certain beneficial functions to contribute to the health of the host, leading to the vulnerability of the host to diseases, e.g., CRC.

In fact, recent discoveries on the genetic diversity and population structure of *E. coli* have encouraged investigators to associate certain phylogenetic groups or clonal complexes with human diseases [23, 83–85]. Such “intra-” *E. coli* diversity has mostly been treated as genetic differences within a “single” bacterial species. However, at least in views of bacterial genetics and pathogenicity, the evolutionary divergence between *E. coli* K12 and O157:H7 should have separated them into entirely different bacteria, which, with the former being a harmless commensal and the latter a deadly pathogen [56, 86], may have over a thousand genes different between them. In the analysis of the fresh *E. coli* isolates in this study, we profiled the genetic variations among them and found that the levels of overall genetic divergence among them were similar to that of *E. coli* K12 and O157:H7. The large numbers of novel genes specific to the individual *E. coli* genotypes would render the bacteria different abilities or preference to adapt to a broad range of micro-niches in the gut environment. Additionally, the genetic separation of the *E. coli* bacteria by clear-cut boundaries strongly indicates their non-overlapping environmental settings in the intestinal lumen of a host. Indeed, the co-existence of genetically diverse *E. coli* lineages in healthy people would be best explained by their inability or unnecessity to purge each other, very possibly due to their difference in resource requirement. Therefore the very low diversity of the *E. coli* isolates from CRC patients in sharp contrast with the high diversity of the bacteria in the healthy individuals is indicative of the health significance of *E. coli* diversity to the host. Notably, the *E. coli* isolates from the CRC patients suppressed the growth of *E. coli* isolates from healthy controls when the nutrients were limited, suggesting their greater growth capabilities, which may in turn result in unhealthy microenvironments in the intestine and facilitate the carcinogenesis of the intestinal tissues.

It is a very interesting observation that the *E. coli* diversity increased with age, which may reflect a general scenario of dynamic *E. coli* diversification during the life time of the host. But how is the diversity reached?

Bacteria acquire genetic novelty and diverge by two major mechanisms, including incorporating large exogenous DNA segments, e.g., prophages, genomic islands, etc., and accumulating nucleotide substitutions, with the former being mostly acute to divert the evolutionary direction and the latter usually chronic to make gradual genomic ameliorations in a chronological way [31, 87, 88]. Comparisons between *E. coli* K12 and O157:H7 as well as among the genotypes indicate that the acquisition of large exogenous DNA segments is highly frequent in evolution and the events may happen almost instantly. However, the final acceptance of the acquired DNA segments may take time for genomic ameliorations toward eventual adaptation. If one assumes that bacterial genomic ameliorations take place at similar rates among different bacteria [89, 90], the different *E. coli* lineages, with genetic distances between them being similar to that as between *S. typhi* and *S. typhimurium* (see Fig. 3), would be estimated to have a divergence time of 35–50 thousand years [91]. Therefore, the striking diversity of commensal *E. coli* revealed in this study would be a result of gradual colonization of a host by pre-existing *E. coli* lineages, rather than de novo creation of nascent *E. coli* lineages through divergence. Indeed, the phylogenetic divergence would require evolutionary times that are much longer than the lifespan of a human host. The colonization process may take certain lengths of time, which may partly explain why *E. coli* in people with advanced ages may have higher genomic diversity than in junior people. Although high genomic diversity of the *E. coli* populations is positively correlated with health status, we currently have no direct evidence yet to show whether the low *E. coli* diversity in the CRC patients might be the consequence or an etiologic factor of the disease.

## Conclusions

Commensal *E. coli* are diverse and the diversity increases in the healthy hosts with age from children through young adults to longevity seniors, suggesting that the coexistence of multiple *E. coli* lineages may help create and maintain a microbial environment that is beneficial to the host. The diversity is due to a high number of discrete phylogenetic clusters rather than continual genetic variations spanning a wide spectrum of the *E. coli* bacteria. *E. coli* isolates from CRC patients had growth advantages over those from healthy individuals, suggesting their potential pathogenic roles.

## Supplementary information

**Additional file 1: Table S1.** Percentages of common genes with identical nucleotide sequences among the fresh *E. coli* isolates from healthy individuals.

**Additional file 2: Table S2.** Profile of genes in the 25 *E. coli* isolates in common with *E. coli* K12 MG1655.

**Additional file 3:** **Table S3.** Profiles of genes not present in K12 MG1655 but present in one or more of the 25 *E. coli* isolates.

**Additional file 4: Figure S1.** Genomic comparison of sequenced fresh *E. coli* isolates with strain K12 MG1655 to show the presence or absence of K12 MG1655 in the sequenced fresh *E. coli* isolates. Genes present in any of the sequenced fresh *E. coli* isolates but not in K12 MG1655 are listed in Supplementary Table 3.

## Data Availability

The datasets used and/or analyzed during the current study are available from the corresponding author on request.

## Abbreviations

CRC: Colorectal cancer
Genotypes: Genome types
NJ method: Neighbor-joining method
PFGE: Pulsed field gel electrophoresis
